# The Effect of Sleep Deprivation on Cardiac Function and Tolerance to Ischemia-Reperfusion Injury in Male Rats

**DOI:** 10.5935/abc.20150137

**Published:** 2016-01

**Authors:** Sajad Jeddi, Amir Nezami Asl, Alireza Asgari, Asghar Ghasemi

**Affiliations:** 1 Endocrine Physiology Research Center, Research Institute for Endocrine Sciences, Shahid Beheshti University of Medical Sciences, Tehran - Iran; 2 Aerospace Medicine Research Center, Medical Faculty of Aerospace and subaquatic Medicine, AJA Medical Sciences University, Tehran - Iran

**Keywords:** Sleep Deprivation, Sleep Initiation and Maintenance Disorders;, Ventricular Dysfunction, Myocardial Ischemia, Myocardial Reperfusion Injury, Nitric Oxide, Rats

## Abstract

**Background:**

Sleep deprivation (SD) is strongly associated with elevated risk for cardiovascular disease.

**Objective:**

To determine the effect of SD on basal hemodynamic functions and tolerance to myocardial ischemia-reperfusion (IR) injury in male rats.

**Method:**

SD was induced by using the flowerpot method for 4 days. Isolated hearts were perfused with Langendorff setup, and the following parameters were measured at baseline and after IR: left ventricular developed pressure (LVDP); heart rate (HR); and the maximum rate of increase and decrease of left ventricular pressure (±dp/dt). Heart NOx level, infarct size and coronary flow CK-MB and LDH were measured after IR. Systolic blood pressure (SBP) was measured at start and end of study.

**Results:**

In the SD group, the baseline levels of LVDP (19%), +dp/dt (18%), and -dp/dt (21%) were significantly (p < 0.05) lower, and HR (32%) was significantly higher compared to the controls. After ischemia, hearts from SD group displayed a significant increase in HR together with a low hemodynamic function recovery compared to the controls. In the SD group, NOx level in heart, coronary flow CK-MB and LDH and infarct size significantly increased after IR; also SD rats had higher SBP after 4 days.

**Conclusion:**

Hearts from SD rats had lower basal cardiac function and less tolerance to IR injury, which may be linked to an increase in NO production following IR.

## Introduction

Cardiac ischemia is the principle cause of human death worldwide^1,2^ and its rate is rising because of co-morbid diseases, such as diabetes and obesity, and also aging.^3^ Cardiac ischemia is often induced by the occlusion of coronary arteries and while reperfusion can salvage the ischemic heart from death, it can induce side effects, known as ischemia‑reperfusion (IR) injuries.^4^

Sleep is a vital regulator of cardiovascular function, both in the physiological state and in disease conditions.^5^ Previous cohort and case-control studies have indicated that sleep disorders are related to an increased prevalence of cardiovascular disease and even an independent risk factor for the development of that disease.^6,7^ Sleep disorders exert harmful effects on a variety of systems with obvious changes in the endocrine, metabolic and immune pathways that are related to unfavorable health outcomes, including diabetes, hypertension, and obesity that are documented to contribute to the development of cardiovascular disease.^6,7^

Nitric oxide (NO) is synthesized by NO synthase enzymes in the heart and plays a vital role in cardiac functions. Despite the evidence highlighting the role of ischemia in a rise in NO production,^8,9^ no studies have yet examined the changes in the NO content in the hearts of sleep deprivation (SD) rats and its contribution to IR injury. Moreover, to the best of our knowledge, there is no study addressing the effects of SD on basal cardiac function and cardiac tolerance following IR injury. Therefore, the aim of this study was to determine the effect of SD on basal hemodynamic functions and tolerance to myocardial IR injury in male rats. In addition, changes in NO metabolites (NOx) following IR injury were also assessed.

## Methods

### Animals

In this study, 16 male Wistar rats (2-month old) were obtained from laboratory animal house of the Research Institute for Endocrine Sciences (RIES), Shahid Beheshti University of Medical Sciences. All animals were housed in an animal room with temperature and light controlled conditions (22 ± 2ºC 12/12-h dark-light cycle), and had free access to food and water at all times.

The proposal of this study was approved by the Institutional Animal Care and Use Committee (IACUC) of the RIES (Permit Number: 12 EC RIES 93/11/27), Shahid Beheshti University of Medical Sciences (Tehran, Iran).

### Induction of REM sleep deprivation

Male rats were deprived of rapid-eye-movement (REM) sleep for 96 hours. Rapid-eye-movement SD was induced by using the flowerpot technique.^10^ Briefly, rats were placed on an inverted pot (platform of 7-cm diameter) surrounded by water reaching to a level of 1cm under the bottom of the pot. Tank's water was refreshed every morning. Prior to depriving rats of sleep, they were trained for 1-2 hours to stay steady on the platform and not to fall into the water. After training, on the day of experiment, they were taken from their home cage and placed individually in the water tank.

For experiments, animals were randomly divided into 2 groups (n = 8 each): Control and SD.

### Isolated heart preparation

All animals were anesthetized with an intraperitoneal injection of ketamine and xylazine (50 mg/kg and 10 mg/kg, respectively). Once fully anesthetized, the thorax was opened and the hearts of control and SD rats were rapidly excised and embedded in an ice-cold perfusion buffer. The aortas were then cannulated and the whole preparation mounted in the Langendorff perfusion apparatus and perfused through the aorta with a Krebs-Henseleit solution containing (mM/L): NaCl 118, NaHCO_3_ 25, KCl 4.7, MgCl_2_ 1.2, CaCl_2_ 2.5, KH_2_PO_4_ 1.2, and glucose 11 at a constant pressure (75 mm Hg) and pH of 7.4. The Krebs solution was gassed with a combination of 95% O_2_ and 5% CO_2_ at 37ºC. All isolated hearts were stabilized for 20 minutes with the purpose of obtaining baseline data. After stabilization, hearts were subjected to global ischemia for 30 minutes followed by reperfusion for 45 minutes. Left ventricular hemodynamic parameters were measured via a latex balloon inserted in the left ventricle. The balloon capacity was adjusted to create 5-10 mm Hg of end-diastolic pressure in all hearts by filling it with water. Hemodynamic parameters [left ventricular end-diastolic pressure (LVEDP), heart rate (HR), left ventricular developed pressure (LVDP), and the maximum rate of increase and decrease of left ventricular pressure (±dp/dt)] were digitized and recorded by a data acquisition system (Power Lab, AD Instrument, Australia).

### Measurement of NOx

Following 45-min reperfusion, samples of left ventricular tissue were immediately frozen in liquid nitrogen and stored at -80ºC. NOx contents in heart homogenates were determined using the Griess method^11^. Briefly, after homogenization of samples in phosphate-buffered saline (1:5, w/v), the homogenates were centrifuged at 15,000 *g* for 20 min at 4ºC. The supernatants were removed from the homogenates and were deproteinized by addition of zinc sulfate (15 mg/mL). Tissue samples, 100 µL of the supernatant were added to a microplate well, and 100 µL vanadium (III) chloride (8 mg/mL) were added to each well (for reduction of nitrate to nitrite), followed by addition of 50 µL sulfanilamide (2%) and 50 µL NEDD (N-(1-naphtyl) ethylendiamine dihydrochloride) (0.1%). After 30-min incubation at 37ºC, the absorbance was read at 540 nm using the ELISA reader (BioTek, Powerwave XS2). NOx concentrations in heart homogenate samples were measured from the linear standard curve established by 0-100 µmol/L of sodium nitrate. Heart NOx levels are presented as µmol/L. Intra-assay coefficient of variation was 4.9%.

### Measurement of infarct size

At the end of the reperfusion period, infarct sizes were determined as described previously. The frozen heart samples were cut into thin slices (2-3 mm) and were incubated for 10 minutes in 1% of 2,3,5-triphenyltetrazolium chloride in phosphate buffer solution 20 mM/L, pH 7.4 at 37ºC. The slices were immersed in 10% formalin for 24 hours to identify viable myocardium as red stained, easily discriminable from unstained necrotic tissue. The sections were then photographed using a digital camera (Samsung, Japan, version DV101). The infarct size was measured by Photoshop CS6 software (version 13) and expressed as percentage of the total area.^2^

### Measurement of creatine kinase (CK) and lactate dehydrogenase (LDH)

Samples of coronary flow were collected for 5 minutes after start of reperfusion for measuring the myocardial enzyme leakage, CK-MB and LDH.^12^ The CK-MB and LDH levels in the coronary effluent were determined by spectrophotometric method via CK-MB kit and LDH kit (Pars Azmoon, Iran) and the results were expressed as U/L.

### Statistical analysis

All values are expressed as means ± SEM. Statistical analysis was performed using SPSS software (SPSS, Chicago, IL, USA; version 20). The Shapiro-Wilk test was used to check the normality of study data, and then parametric or non-parametric tests were used to analyze normal or non‑normal data distribution, respectively.^13^ Therefore, repeated measurement analysis of variance (ANOVA) was used to compare hemodynamic parameters (LVDP, LVEDP, ±dp/dt, and HR) in different times. The Student *t* test was used to compare blood pressure, infarct size, heart NOx levels and coronary flow CK-MB and LDH between control and SD groups. Two-sided p-values < 0.05 were considered statistically significant.

## Results

**Figure 1:**
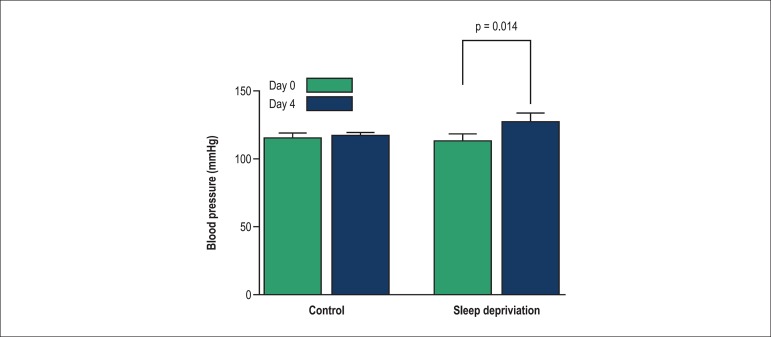
Comparison of changes in systolic blood pressure in control and sleep deprivation groups. Values shown as mean ± SEM.

After 96 hours of SD, the rats showed a significant increase in systolic blood pressure (SBP) compared to the control group (Figure 1).

**Figure 2:**
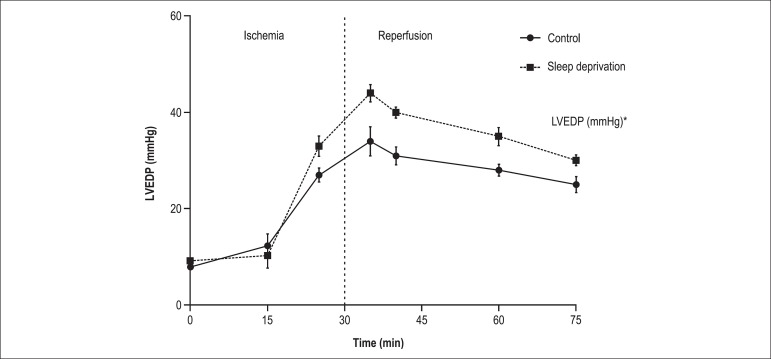
Change in left ventricular end-diastolic pressure (LVEDP) (ischemic contracture) during experiment. Values shown as mean ± SEM (n = 8 rats)

**Figure 3:**
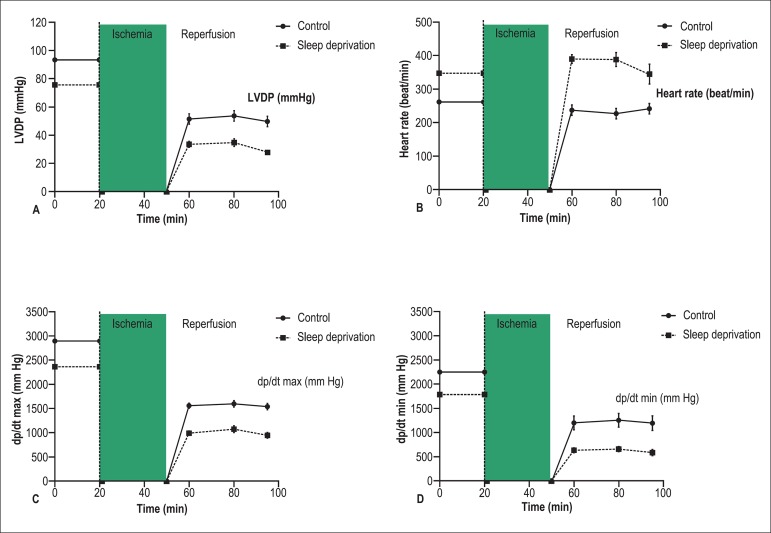
Recovery of cardiac function after ischemia-reperfusion injury. A. Left ventricular developed pressure (LVDP); B. Heart rate; C. Peak rates of positive changes in left ventricular pressure (+dp/dt); D. Peak rates of negative changes in left ventricular pressure (- dp/dt). Values shown as mean ± SEM (n = 8 rats)

Effects of SD on hemodynamic parameters in isolated heart during the stabilization and IR periods are shown in Table 1, Figure 2, and Figure 3. As seen, during the stabilization period, hearts from SD rats had significantly lower baseline LVDP and ±dp/dt, and higher HR (p < 0.05) compared to the control group (Table 1). When there was ischemia, the LVDP, ±dp/dt, and HR rapidly decreased and stopped in the isolated hearts. In both groups, LVEDP was gradually increased during the 30 minutes of ischemia, however the SDgroup displayed a significant increase (p < 0.05) compared to the control group (Figure 2). Compared with the control group, post-ischemic LVDP and ±dp/dt were significantly lower and HR was higher in the SD group (p < 0.05) (Figure 3-A, B, C, and D).

**Table 1:** Cardiac function at the stabilization period (baseline data)

	Control	Sleep deprivation	p value
LVDP*	93.14 ± 4.9	75.5 ± 3.5	0.035
Heart rate*	262.2 ± 11.1	347.5 ± 19.7	0.003
dp.dt max*	2897.1 ± 75.1	2367 ± 222.7	0.021
dp.dt min*	2555.3 ± 227.6	1788.8 ± 105.9	0.046

Data shown as mean ± SEM. LVDP: Left ventricular developed pressure; ± dp/dt: peak rates of positive and negative changes in left ventricular pressure;

* p < 0.05.

**Figure 4:**
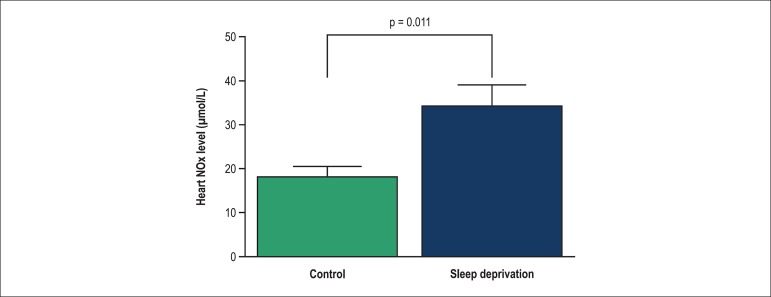
Change in NOx in control and sleep deprivation groups in heart after ischemia. Values shown as mean ± SEM (n = 8 rats)

IR induced a marked increase in the heart NOx level in the SD group compared with the control group (Figure 4).

**Figure 5:**
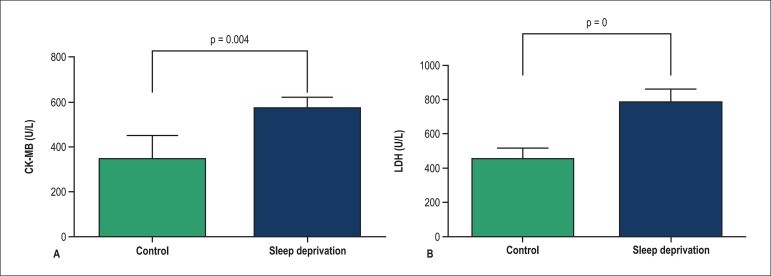
Change in coronary flow CK-MB (A) and LDH (B) in control and sleep deprivation groups in start of reperfusion. Values shown as mean ± SEM (n = 8 rats)

Coronary flow CK-MB and LDH levels were significantly (p < 0.05) higher in the SD group compared to the control group (p < 0.05) (Figure 5).

**Figure 6:**
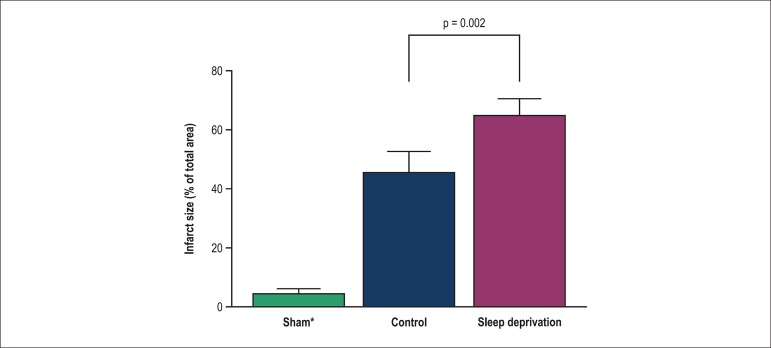
The alterations of infarct size in heart of control and sleep deprivation rats. Values shown as mean ± SEM (n = 8 rats)

Sleep deprivation significantly increased the infarct size in SD rats compared to control rats (Figure 6).

## Discussion

The results of this study showed for the first time that SD for 96 hours induces negative inotropic and chronotropic effects on the hearts isolated from male rats and increases the IR-induced injury in those hearts, which may be linked to increasing the NO production after IR.

In this study, SBP was higher in the SD group compared to controls. Several studies have found that experimental SD leads to increased blood pressure, which is in line with our findings. Neves et al^14^ have shown that rats with 72-hour REM SD had higher blood pressure compared with controls. Joukar et al^15^ have indicated that SD resulted in a significant increase in blood pressure in rats by using the similar method. De Mesquita and Hale^16^ have also shown that SD in rats led to increasing blood pressure following 114 hours of REM SD. As stated by others, blood pressure could have increased during SD due to either increased sympathetic outflow to the heart or periphery, or changes in baroreflex sensitivity to a higher level, or a combination of both.^6,17,18^

Pre-ischemic and reperfusion HR were higher in the SD group than in controls, which is consistent with the reports by others. Sgoifo et al,^19^ Carvalho et al,^20^ and Almeida et al^21^ have reported that SD rats exhibited an increased HR and were vulnerable to ventricular arrhythmias. The mechanism of action of SD on HR during perfusion and reperfusion is not completely understood. In this regard, studies have shown that SD increases HR through various mechanisms. One possible cause for the significant increase in HR in SD rats before and after ischemia may have resulted from the immobility stress induced by the flowerpot technique. Sleep deprivation affects the basal activity of the stress system by increasing the corticosterone and adrenocorticotropic hormone concentrations and the subsequent response to an acute suppression in the cardiac autonomic and hypothalamic-pituitary-adrenal axis.^19,22^

We recorded lower baseline LVDP and ± dp/dt in the SD group, which indicate that SD could affect the normal heart function. The exact mechanisms of the SD effects on baseline cardiac functions are not fully understood and need further elucidation, but SD has been suggested to indirectly increase cardiovascular disease by increasing inflammation and cortisol secretion, altering growth hormone metabolism and changing the circulating levels of ghrelin and leptin.^7^ In addition, we showed for the first time that, after ischemia, hearts from the SD group displayed a significantly low hemodynamic function recovery compared to controls, indicating that these hearts are susceptible to the IR injury, as confirmed by the increase in the LDH and CK-MB levels in the coronary effluent and the rise in the infarct size.

In this study, consistent with other studies on disease states, such as hyperthyroidism,^9^ diabetes^23^ and maternal hypothyroidism,^2^ ischemia increased the CK-MB and LDH levels and the infarct size in the SD group, which showed heart cell necrosis. The heart cells contracted during long or severe ischemia and start of reperfusion, causing mechanical stiffness and tissue necrosis. Contracted cells put pressure on neighboring cells and cause their decomposition and development of contraction, leading to widespread necrosis.^12,24^

In this study, the capacity of the balloon in the left ventricle was fixed during ischemia and reperfusion, so the increase in LVEDP resulted from stiffness in the left ventricular wall or ventricular contracture. Thus, SD could be said to increase contracture and accordingly to significantly increase the subsequent necrosis in the IR period in the heart of SD rats. Increasing Ca^2+^ concentration in heart cells and ATP depletion have been reported to be important factors in ischemic contracture.^25^

The function of NO in the cardiovascular system after ischemia has not been fully enlightened. A few studies have considered a negative role for NO in myocardial IR injury, whereas others have reported a protecting role. Recent studies have shown that the NO level of myocardial cells is in a low range at baseline and increases during IR injury. While a low range increase in NO production may be cardioprotective, a vast increase appears to be injurious. At high levels, NO reacts with superoxide and produces peroxynitrite, which is a highly toxic agent that could induce apoptosis in heart cells.^8,9^ Our results show that SD increases IR-induced injuries in rat hearts, which may possibly be linked to elevated NO levels after ischemia. Thus, it could be hypothesized that SD may lead to reperfusion injury by increasing the NO production.

Regarding the limitations of this study, we used global ischemia, which has been commonly used in the Langendorff-perfused heart model. However, the use of the regional ischemia model has been reported to be clinically more relevant.^26^ In addition, our results are limited to male rats, while SD affects cardiac functions in both sexes.^27^

## Conclusion

Based on our findings we conclude that the hearts of SD rats had lower basal cardiac function and less tolerance to the IR injury, confirmed by the increase in LDH and CK-MB levels in coronary flow. In addition, the rise in the infarct size may be due to an increase in the NO production after IR.
